# Local dynamics within the glass transition domain

**DOI:** 10.1038/s41598-019-45933-2

**Published:** 2019-07-03

**Authors:** François Godey, Alexandre Fleury, Armand Soldera

**Affiliations:** Department of Chemistry, Centre Québécois sur les Matériaux Fonctionnels, Université de Sherbrooke, Sherbrooke, (Québec) J1K 2R1 Canada

**Keywords:** Computational methods, Glasses, Polymers

## Abstract

The glass transition of an amorphous material is a fundamental property characterized by an abrupt change in viscosity. Its very knowledge was a conundrum as no satisfying theory existed at the molecular level. We herein relate this complex phenomenon to events occurring at the molecular scale. By studying conformational transitions in the carbon-chain polymer of polyethylene, we clearly establish a relation between local dynamics and the classical dihedral potential energy diagram of a carbon-carbon bond. This methodology is applied to a carbon-chain polymer with a side-group, polystyrene. A direct link is proved between activation energy and glass transition temperature. This work thus provides the cornerstone for linking molecular structure to macroscopic polymer properties, and in particular, the glass transition temperature.

## Introduction

Numerous systems such as polymers exhibit a glass transition. As the temperature is decreased they turn from an amorphous state of low viscosity to a supercooled liquid with very high viscosity^1^. This transition is characterized by the glass transition temperature (*T*_*g*_). Despite being known for a very long time it remains a thrilling domain of research as its very nature is not fully solved^2^. The main reason is that no experiments or theories can grasp its entire domain of dynamic ranging from nanoseconds to years (ageing). These recent years, molecular simulation became an additional and powerful technique to complement existing data or current theories^3^. Among the different simulation tools, molecular dynamics simulation is of special interest as it can probe local dynamics. In 1988, Rigby and Roe, in their seminal work, showed that *T*_*g*_ can be extracted for polymers from full-atomistic simulation^4^. Since then, a huge number of simulations dealing with the glass transition of polymers have been reported^5–7^. However, despite nice linear relationship between simulated and experimental *T*_*g*_, as our group showed for instance^8^, the extremely rapid simulated cooling rate, in order of 10^11^ times more rapid than usual experiments, raise questions on its meaning. We thus propose, in this study, to shed some light on this issue by exploring the energy landscape, a powerful approach to deal with the glass transition. Consequently, a chemical picture of this tricky transition will emerge.

For decades, *T*_*g*_ was defined by the temperature at which the viscosity reaches 10^13^ poise^1,9^. Nowadays, Differential Scanning Calorimetry (DSC) remains the most common technique to measure it by detecting change in the heat capacity of the sample occurring at the glass transition^10^. Dynamical Mechanical Analysis (DMA) is also greatly employed since it measures differences in viscoelasticity with temperature. Regardless the experimental technique, the temperature domain associated with the glass transition is in order of 3 to 5 K^10^. The most used simulation method to locate *T*_*g*_ is the dilatometry technique^4^. It consists in reporting the specific volume with respect to the temperature. The intersection between two linear fits at low and high temperatures gives *T*_*g*_. However, we recently showed that due to the extremely rapid cooling rate, molecular dynamics (MD) simulation leads to a spreading of the glass transition domain, in order of 160 K^11^. It was shown that the heat capacity and the coefficient of thermal expansion vary gradually between two limit temperatures, the lower $({T}_{g}^{l})$ and the upper $({T}_{g}^{u})$ transition temperatures. A comparison was then proposed with ultra-fast camera leading to the so-called overcrancked effect where molecular features should be unveiled. Accordingly, behind the measurement of *T*_*g*_ from MD, the meaning of these two extra temperatures during the glass transition process must also be investigated. To unravel their significance, computing the activation energy (*E*_*a*_) is a very attracting avenue as it depicts local dynamics^12,13^. Moreover, it can be related to the energy landscape introduced by Angell^14^, and developed by Debenedetti and Stillinger^15^, one of the prevailing current theories to untangle the glass transition mystery.

In the energy landscape picture, a glass is trapped in a specific basin. The height of its barrier is directly related to the glass properties^16^. However, despite the very interesting outlook it brings, its depiction remains a challenging task. The activation energy corresponds to the energy necessary to go from one potential energy minimum to another inherent structure^15^. In polymers, it is numerically computed from an Arrhenius plot where frequencies of transitions between different rotameric states are reported with respect to the inverse of temperature^13,17–19^. Nevertheless, the very interpretation of *E*_*a*_ stemming from MD simulation was source of interrogation^20^. More specifically, an Arrhenius depiction of such relaxation remains surprising. It was suggested to be in relation with the computed potential energy barrier computed for the rotation of a single bond, in addition to be directly linked to *T*_*g*_^20^. As pointed out by Boyd and Smith, “The resolution of these questions follows and leads to considerable insight into the nature of glass formation in polymer melts”^20^. We thus propose to address the relationship between *E*_*a*_ and the temperatures associated with the glass transition, *T*_*g*_, and the two temperature limits, through a description of a relevant portion of the potential energy landscape^15^. For this, two polymers are considered, one with the simplest architecture, polyethylene (PE), and one with a side-chain, polystyrene (PS). We then argue that an atomistic representation of the glass transition emerges.

## Results and Discussion

**Figure 1:**
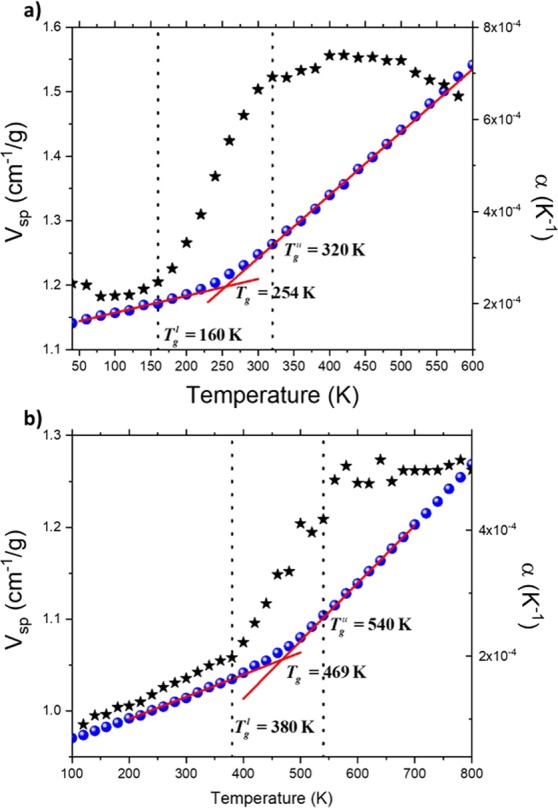
Specific volume () and thermal expansion coefficient (★) with respect to the temperature (K) for PE (**a**), and PS (**b**). The glass transition domain is limited by vertical dot lines at 160 K ${T}_{g}^{l}$ and 320 K ${T}_{g}^{u}$, and 380 K ${T}_{g}^{l}$ and 540 K ${T}_{g}^{u}$ for PE **(a**) and PS (**b**), respectively.

To get numerical value of *T*_*g*_, the simulated dilatometry technique is employed^4^. The specific volume is reported with respect to the temperature, as it is shown in Fig. 1a and b, for PE and PS, respectively. Values of *T*_*g*_ are 254 K and 469 K for PE and PS, respectively. The actual display of a discontinuity in the linear behavior of the specific volume with respect to the temperature is the sign of a change in the molecular behavior. However, as we recently reported, due to the very rapid cooling rate (in order of 10^11^ times more rapid that the experimental rate), this discontinuity is spread between two temperatures, a lower ${T}_{g}^{l}$ and upper ${T}_{g}^{u}$, as revealed by the behavior of the thermal expansion coefficient (Fig. 1), the heat capacity^4^, or the bulk modulus^21^. It was argued that MD simulation acts as an ultra-fast camera leading to overcranking effects, enabling very slow-motion to be captured. This spread occurs between 160 K and 320 K, for PE (Fig. 1a), and 380 K and 540 K, for PS (Fig. 1b). For both polymers (Fig. 1), no changes in the behavior of the coefficient of thermal expansion are detected at *T*_*g*_. The local dynamics is then investigated in the whole glass transition domain bounded by ${T}_{g}^{l}$ and ${T}_{g}^{u}$. For this, the activation energy associated with the backbone motion is computed.

To establish the link between *E*_*a*_ extracted from an Arrhenius diagram, and the values of energies in the dihedral potential energy curve associated with the carbon-carbon bond, a reference is needed. We propose to use the following equation:

$${E}_{dihedral}(\varphi )={c}_{1}[1+\,\cos (\varphi -\pi )]+{c}_{2}[1-\,\cos (2(\varphi -\pi ))]+{c}_{3}[1+\,\cos (3(\varphi -\pi ))]$$

**Figure 2:**
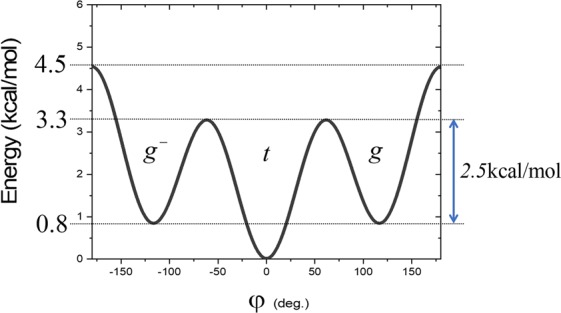
Dihedral potential energy for a carbon-carbon bond^21^.

With the *trans* state is at 0 deg, and *c*_1_, *c*_2_, and *c*_3_, are coefficients whose values are 0.7055, −0.1355, and 1.5722 kcal/mol respectively^19,22^. The energetical diagram is displayed in Fig. 2. Three reference energies are indicated therein: energy of the *gauche* (*g* or *g-*) state, 0.8 kcal/mol, barrier heights to pass from the *trans* to *gauche* states, 3.3. kcal/mol, and to exchange the *gauche* states, 4.5 kcal/mol. The barrier height to go from a *gauche* state to the *trans* state is also indicated. Comparison with *E*_*a*_ stemming from the description of the polymer local dynamics can now be proceeded.

**Figure 3:**
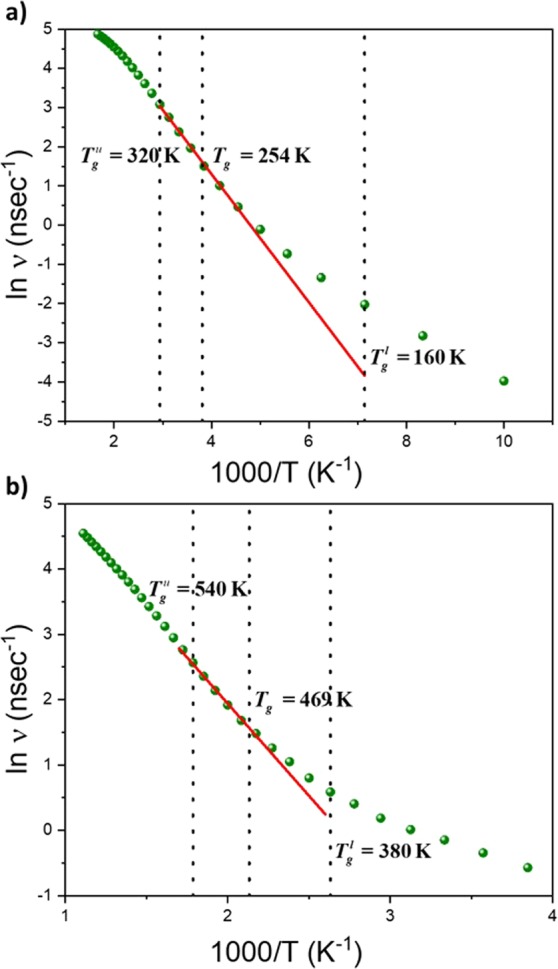
Arrhenius diagram for the all backbone transitions () for PE (**a**), and PS (**b**).

To compute *E*_*a*_, the number of transitions between two rotameric states must first be established. It must be pointed out that all the reported data correspond to averages over eight configurations. The way a transition is registered was discussed in previous publications and summarized in the simulation methods paragraph^13^. Arrhenius diagrams for all transitions along the backbone chain in PE and PS are shown in Fig. 3.

**Figure 4:**
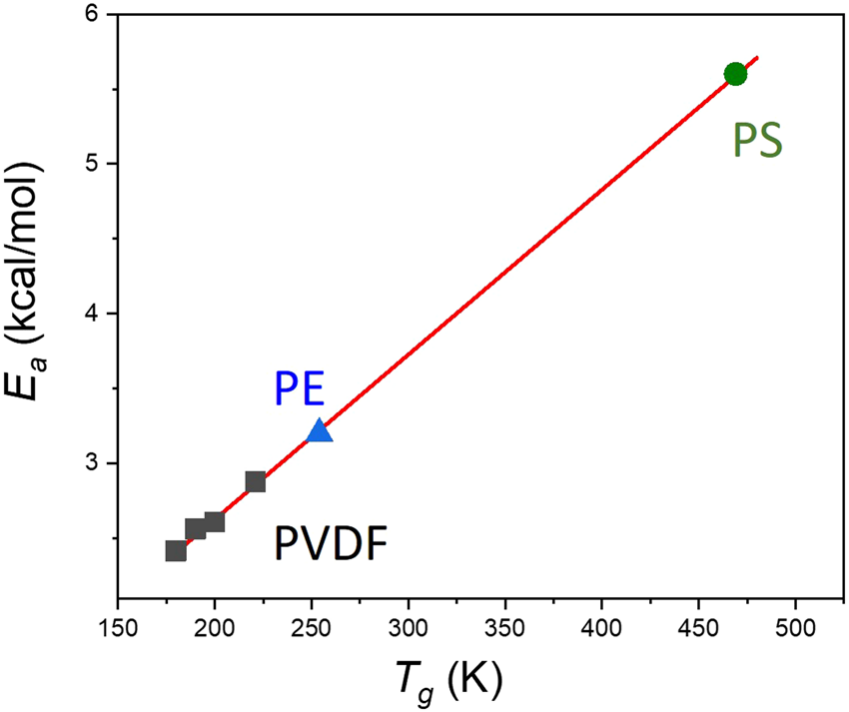
Activation energy (*E*_*a*_ in kcal/mol) with respect to the *T*_*g*_ of PVDFs (■)^13^, PE () and PS ().

*E*_*a*_ is directly extracted from the slope in the Arrhenius diagrams displayed in Fig. 3. The linear regression is made between *T*_*g*_ and ${T}_{g}^{u}$. A value of 3.2 kcal/mol for *E*_*a*_ for PE is supported by previous simulations and experimental data^23^. Moreover, this value was usually suspected to be correlated with the actual potential energy barrier necessary for a bond to go from the *trans* to *gauche* states, as shown in Fig. 2 ^24–26^. It remains surprising that cooperative effects can be integrated in a value that corresponds to the simple barrier height between *trans* and *gauche* (Fig. 2). However, for PS, *E*_*a*_ is equal to 5.6 kcal/mol (Fig. 3b). This value should transcribe the cooperativity between side-chain and backbone motion as it is different from the potential energy height of a carbon-carbon bond (Fig. 2). It is in agreement with published data of 6 kcal/mol^27^. Nevertheless, these values of *E*_*a*_ stemming from MD have been claimed by several authors including ourselves to be correlated with *T*_*g*_^13,25^. These data have thus been inserted in the graph of *E*_*a*_ (*T*_*g*_) previously derived from polyvinyldifluoride (PVDF) and its derivatives with different percentages of regioisomerism defects, in Fig. 4 ^13^. The additional data fit perfectly the linear regression arising from the previous data. The new linear regression is:

$${E}_{a}(\mathrm{kcal}/\mathrm{mol})=0.01\cdot {T}_{g}(K)+0.44\,\mathrm{kcal}/\mathrm{mol}$$

with R^2^ of 99.9%. The only difference with previous fitting equation lies in the ordinate at the origin, before $0.64\,\mathrm{kcal}/\mathrm{mol}$. Such a fit confirms the intimate relationships between *T*_*g*_ and *E*_*a*_.

The linear relationship between *E*_*a*_ and *T*_*g*_ (Fig. 4) is of the utmost importance as it gives a molecular perspective of the nature of the glass structure. Accordingly, knowing *E*_*a*_ for each kind of backbone fragments of a polymer can lead to the computation of *T*_*g*_ stemming from simulated dilatometry, as we showed for E-PTFE^13^. Moreover, since *E*_*a*_ depicts local dynamics, it can be argued that *T*_*g*_ from MD is relevant to describe the backbone motion underlying the glass transition. 5.6 kcal/mol thus represents the activation energy associated with the backbone motion of the PS chain. However, in the case of PE, *E*_*a*_ is deduced from the simple barrier height between *trans* and *gauche* rotameric states (Fig. 2). This simple picture does not ultimately estimate cooperative effects^28^. In fact, transitions reported in Fig. 3 include all kinds of transitions whatever the transition involves cooperative transition or local changes in the position of neighboring atoms. Separation between these two categories must be carried out.

**Figure 5:**
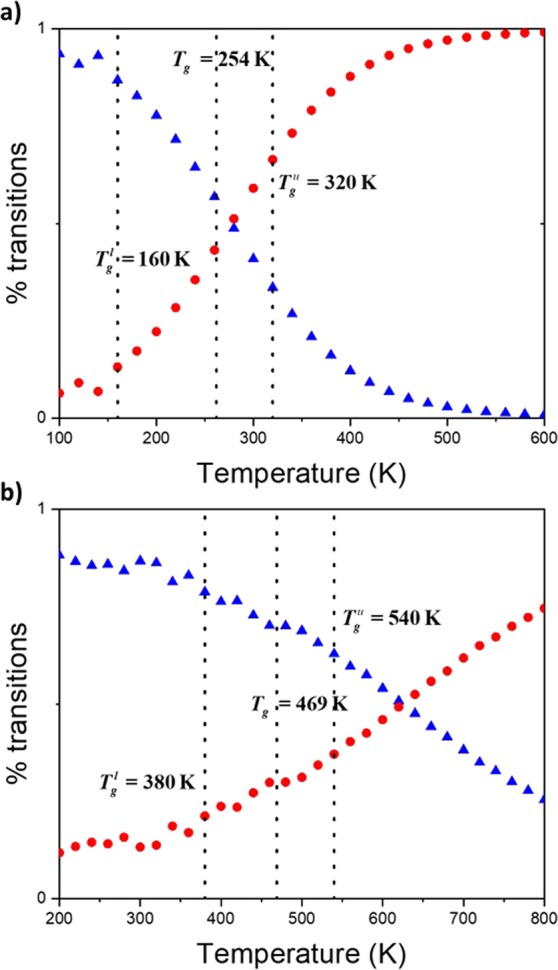
Percentage of cooperative () and isolated transitions () with respect to temperature for PE (**a**) and PS (**b**). Vertical lines representing *T*_*g*_, ${T}_{g}^{l}$ and ${T}_{g}^{u}$ are also shown.

Local dynamics is mainly governed by two types of motion: i) rotational transitions of backbone bonds from one rotameric state, i.e. *trans*, *gauche*, and *gauche-*, to another, and ii) librational motions about rotameric minima and fluctuations in bond lengths and bond angles^29^. Intramolecular jumps in bond rotations associated with the backbone motion are actually due to librational motions^29,30^. However, configurational transition referred as triggering transition^31^, can take place without resulting in large displacement of the whole polymer chain by two main mechanisms^12^. (1) Cooperative transitions (CT) correspond to coupling of transition of closely-neighboring bonds along the chain. As a classical example, the crankshaft motion belongs to this category^32,33^. (2) Isolated transitions (IT) do not lead to other dihedral transitions but distort surrounding atoms^31,34^. Such transitions mainly occur at low temperatures^35^. Their particularity is that they do not lead to a chain rearrangement, but a local alteration of atomic positions. Root means square displacements after an IT are shown in Figure of the Supporting Information, at 200 K, and agree published data^35^. Percentages of these two transitions are then shown in Fig. 5 for both studied polymers.

*T*_*g*_ is set following an established protocol (Fig. 1). It does not correspond to a change in the coefficient of thermal expansion behavior. In Fig. 5, it can be observed that neither *T*_*g*_ nor ${T}_{g}^{u}$ are indicative of any changes in the transition percentage. Conversely, ${T}_{g}^{l}$ is the evidence of a change in the chain dynamics. Below this temperature, the number of both transitions remain roughly constant. While for PE, only isolated transitions occur in this domain, some cooperative transitions are detected for PS. The complexity of PS due to the presence of the side-chain should be accountable for their presence. Nevertheless, we can argue that ${T}_{g}^{l}$ coincides with the beginning of cooperative motions as temperature is raised. Cooperative transitions below this transition temperature will not be considered in the rest of the text, in agreement with published data^35,36^. As a result, the stemming activation energies should reflect the entire set of phenomena resulting in the occurrence of the glass transition process, giving insight into the chain dynamics, as outlined by de Gennes^37^. Activation energy for the two kinds of transition can now be investigated.

**Figure 6:**
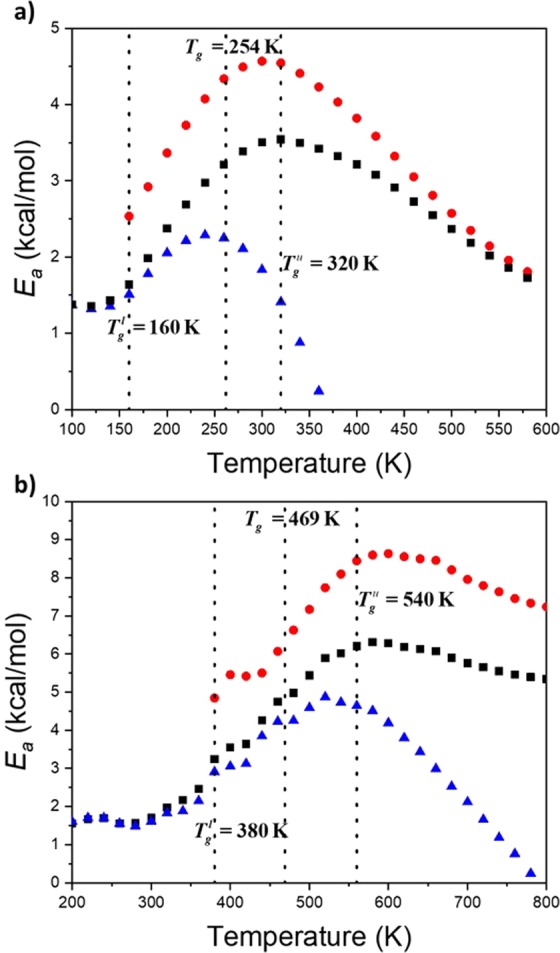
Activation energy (*E*_*a*_) with respect to temperature stemming from the consideration of all backbone transitions, ${E}_{a}^{at}$ (■), cooperative transitions, ${E}_{a}^{ct}$ (), and isolated transitions, ${E}_{a}^{it}$ (), for PE (**a**), and PS (**b**).

Since there is clear variation in physical properties (coefficient of thermal expansion, specific volume and heat capacity) throughout the glass transition domain, between ${T}_{g}^{l}$ and ${T}_{g}^{u}$, the activation energies were computed at each temperature to reveal any changes in the local dynamics. For this, the average of the two slopes associated with a frequency in the Arrhenius diagram is carried out. Three activation energies were thus considered: ${E}_{a}^{at}$ stemming from the consideration of all backbone transitions (Fig. 3), ${E}_{a}^{ct}$ coming from the cooperative transitions, and ${E}_{a}^{it}$ extracted from isolated transitions which perturb only neighboring atomic positions. Their behavior with respect to temperature are displayed in Fig. 6 for both polymers.

General behavior of the different activation energies with respect to temperature (Fig. 6) are comparable for both polymers. The slight difference in the curve shape observed can origin from the presence of a bulky side-group in PS. Discussion on such effect request further development since cooperativity exists between the lateral group rotation and the backbone motion^11^. Values of ${E}_{a}^{at}$ associated with all the transitions are primarily investigated. The actual values stemming from the linear fit in the Arrhenius diagram (Fig. 3) correspond to the average of ${E}_{a}^{at}$ in the graph of Fig. 6 between *T*_*g*_ and ${T}_{g}^{u}$. At this latter temperature, ${E}_{a}^{ct}$ reaches a maximum value. A decrease after this temperature is observed. The reason for this behavior lies in the procedure to count transitions (cf *Simulation methods*). To be recognized as a transition, the new dihedral angle must not change during 1.5 ps. At high temperatures, especially at temperature higher than ${T}_{g}^{u}$, fluctuations increase leading to uncertainties in the definition of a transition. This simulation artifact is no further discussed as the focus is on describing the glass transition domain. At low temperatures, below ${T}_{g}^{l}$, behavior of ${E}_{a}^{at}$ and ${E}_{a}^{it}$ are merged. Only isolated transitions subsist, in agreement with previous conclusions from Fig. 5. This finding also agrees with published data: transitions can occur without any involvement of a rotation in neighboring bonds^31,34^. An interesting statement is that for both polymers, values of ${E}_{a}^{it}$ below ${T}_{g}^{l}$ are equivalent, and equal to 1.5 kcal/mol. However, it is difficult to be certain about the unicity of this value since below ${T}_{g}^{l}$, the number of transitions is limited. Further studies on other polymers are needed. Nevertheless, we argue that it corresponds to the minimum potential energy required to generate one transition between rotameric states along a carbon backbone chain. Such an energy is not sufficient to prompt a transition in another bond (Fig. 2), and thus to engender cooperativity, but it is enough to involve changes in the position of neighboring atoms (Figure in the Additional Information).

**Figure 7:**
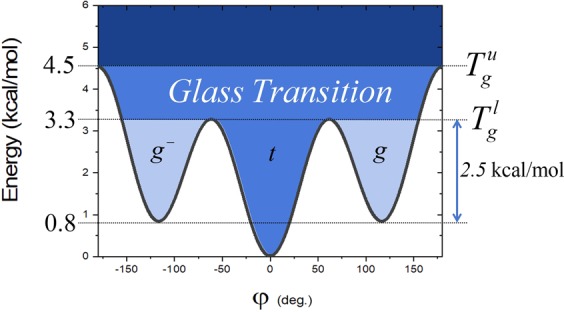
Torsional potential energy with respect to the dihedral angle associated with a C-C bond along the backbone chain with correspondance between energy values and ${T}_{g}^{l}$ and ${T}_{g}^{u}$.

The requested energy to generate a rotameric transition in another bond is ${E}_{a}^{ct}$ at ${T}_{g}^{l}$ where (i) curves of ${E}_{a}^{at}$ and ${E}_{a}^{it}$ split (Fig. 6) and (ii) percentage of CT begins to increase (Fig. 5). At ${T}_{g}^{l}$, the value of ${E}_{a}^{ct}$ is 2.5 kcal/mol and in order of 5 kcal/mol for PE and PS, respectively. For PE, this latter value corresponds exactly to the barrier height between *gauche* and *trans* states (Fig. 2). Based on previous correlation (Fig. 4), we can thus claim that 5 kcal/mol is the potential energy height for *gauche* to *trans* transition in PS along the backbone chain with a phenyl side-group. At ${T}_{g}^{u}$, ${E}_{a}^{ct}$ reaches its maximum value. These values are 4.5 kcal/mol and 8.6 kcal/mol for PE and PS, respectively. For PE, this value corresponds to the total energy barrier height (Fig. 2). From all the information gained from the study of the activation energies, a simple atomistic picture of the glass transition can be retrieved. It is displayed in Fig. 7. The values of the energy are associated with those stemming from PE but can be transferred to PS and other polymers.

In summary, the chemical pictures of the two limit temperatures, and the resulting domains of temperatures (Fig. 7) are as follows:

- $T < {T}_{g}^{l}$: Transitions between rotameric states mainly lead to changes in neighboring atoms position.
- ${T}_{g}^{l}$: Temperature at which there is enough potential energy for transitions between rotameric states to generate rotation in another dihedral angle. This energy corresponds to the barrier height to go from *gauche* to *trans* states.
- ${T}_{g}^{l} < T < {T}_{g}^{u}$: Domain of cooperativity between two bonds, i.e. changes in rotameric states are achieved between two bonds along the polymer chain.
- ${T}_{g}^{u}$: Temperature corresponding to the maximum of the potential energy barrier.
- ${T}_{g}^{u} < T$: Domain of more complex cooperativity.

## Conclusion

Due to very rapid cooling rate, molecular dynamics simulation leads to a spread in the glass transition domain bordered by a lower (${T}_{g}^{l}$) and an upper (${T}_{g}^{u}$) temperatures. The actual enlargement has been seen as an overcranking effect as the coefficient of thermal expansion and the heat capacity vary progressively^11^. However, it challenges the very definition of *T*_*g*_ from atomistic simulation, as it corresponds to an intersection between two linear fits in the dilatometry simulation, not to a change in physical properties. To address this important issue, local dynamics of two polymers, polyethylene (PE) and polystyrene (PS), was investigated thanks to the computation of the activation energy, *E*_*a*_.

By confronting computed activation energies with previously published values of *E*_*a*_ stemming from PVDF derivatives, we clearly demonstrated that *E*_*a*_ is directly related to *T*_*g*_. This value corresponds to a single barrier height in dihedral potential energy diagram. To better grasp the meaning of *E*_*a*_ extracted from the consideration of the backbone transitions, two different kind of transitions were considered: isolated and cooperative transitions. For the former ones, it was shown that they are mainly exhibited at low temperatures in agreement with published studies. It is worth noting that both polymers exhibit the same *E*_*a*_, 1.5 kcal/mol. We argue that this value corresponds to the minimum potential energy required to generate one transition between rotameric states along a carbon backbone chain. However, the unique value for PE and PS cannot be generalized at this point. Further studies are needed. At ${T}_{g}^{l}$, alternative transitions at another bond come into play. The associated *E*_*a*_ is 2.5 kcal/mol which corresponds to the potential energy for a *gauche* state to go in a *trans* state, in the dihedral potential energy diagram. The maximum value is reached at ${T}_{g}^{u}$ where *E*_*a*_ is 4.5 kcal/mol for PE which is the maximum energy in the dihedral potential energy carbon-carbon. For PS, these values are 5 and 8.6 kcal/mol respectively. We thus clearly answer the question raised by Boyd in effect that does *E*_*a*_ behavior appropriate to a single barrier height or whether higher values might arise due to cooperative effect. Accordingly, a simple atomistic picture of the glass transition was shown. This atomic representation should be applied to other systems for which particular behaviour at the glass transition has been reported such as thin films^38^.

## Methods

More details of the whole molecular simulation procedure to get *T*_*g*_ can be found in previous articles^39–41^. Selection of the initial configurations and their relaxation process are crucial. A cell with periodic boundary conditions is constituted by a chain of 250 monomers long for PE, and 125 monomers long for PS. The generation of the chains imbedded in the cell was done through the Self-Avoiding Walk procedure of Theodorou-Suter^42^ and Meirovitch^43^ scanning methods, implemented in the Amorphous_Cell© code, in the Materials Studio environment. 50 configurations were thus first obtained. A first selection was made by considering their radius of gyration not to stray too far from the average value. Second, 8 configurations with the lowest energy are finally selected. A heating-cooling process was then employed to eliminate any endemic stress. Molecular dynamics (MD) in the NPT (i.e., constant number of particles, pressure, and temperature) ensemble was used. The integration of Newton’s equation of motion was performed using the velocity Verlet integration algorithm with a 1 fs integration time step^44^. The Nosé-Hoover thermostat and Parrinello-Rafman barostat algorithms were used to maintain constant temperatures and pressures, respectively^45,46^. The *pcff* force field was chosen. The non-bonded interactions have been computed using the Ewald summation, to take into account long-range effects^3^. All the MD simulations have been carried out using the LAMMPS code^47^. The heating-cooling process consists in a fast heating process (50 K/200 ps) followed by a lower cooling rate (20 K/ns). It has been shown that to get reproducible values of *T*_*g*_, the initial configuration must be in mechanical equilibrium, a “quasi-static” equilibrium state where the stress in the cell balances the internal pressure^48^. A uniform hydrostatic compression is imposed to the system until the internal energy reaches a minimum. During the cooling down process, 20 K/ns, the specific volume is reported with respect to the temperature, for each ensuing configuration. This simulated dilatometry leads to the value of *T*_*g*_. MD of 10 ns are then run at each temperature, and a configuration is saved at each 500 fs.

Details concerning the calculation of *E*_*a*_ can be found in a previous publication. The important initial step is to account for a transition. The method we used can be summarized into 4 key points. The procedure corresponds to a slightly modified Wu’s procedure^18^. (1) Fluctuations in the dihedral angle around the rotameric states are smoothed by a sliding average. (2) In order for a transition to be identified, the difference between the two involved dihedral angles must be greater than 40 deg. (3) The time interval that is requested for a new state along a trajectory to lose memory of its previous state was set at 3 ps. 4) The torsion angle of the new rotameric state must exist for more than 1.5 ps to avoid counting abrupt changes of states.

## Supplementary information

Local dynamics within the glass transition domain
