# Supplementary material for: Local dynamics within the glass transition domain
Source: Sci Rep. 2019 Jul 3;9:9638. doi: 10.1038/s41598-019-45933-2 (PMC6610137; doi:10.1038/s41598-019-45933-2)
Supplement: Supplementary file 1 — Local dynamics within the glass transition domain [file 41598_2019_45933_MOESM1_ESM.docx]

**Additional information**

Local dynamics within the glass transition domain

François Godey, Alexandre Fleury, Armand Soldera*

Department of Chemistry, Centre Québécois sur les Matériaux Fonctionnels, Université de Sherbrooke, Sherbrooke (Québec), J1K 2R1, Canada.

* Corresponding Author: [Armand.Soldera@USherbrooke.ca](mailto:Armand.Soldera@USherbrooke.ca)

Figure. Root means square displacements (RMSD) after isolated transitions at 200 K for PE.
